# Cardiac autonomic ganglion ablation for refractory coronary artery spasm after PCI: a case report

**DOI:** 10.3389/fcvm.2026.1867475

**Published:** 2026-06-09

**Authors:** Fuchao Qu, Shanshan Zhang, Xiufeng Li, Lili Chen, Jiageng Wang, Xiaohua Ma, Chenhui Liu, Yifei Liu, Yuanxin Wang, Leisheng Zhang, Wenwei Yue

**Affiliations:** 1 Department of Cardiovascular Medicine, The Fourth People’s Hospital of Jinan Affiliated to Shandong Second Medical University, Jinan, China; 2 Shandong Provincial Key Medical and Health Laboratory of Blood Ecology and Biointelligence, Jinan Key Laboratory of Medical Cell Bioengineering, Science and Technology Innovation Center, The Fourth People’s Hospital of Jinan Affiliated to Shandong Second Medical University, Jinan, China; 3 Department of Imaging, The First People’s Hospital of Jinan, Jinan, Shandong, China; 4 Department of Pharmaceutics, Integrative Medicine Research Center, Qingdao University, Qingdao, Shandong, China

**Keywords:** catheter ablation of cardiac autonomic ganglia, coronary artery spasm, ergonovine provocation test, heightened vagal tone, Wellens syndrome

## Abstract

A 45-year-old male patient presented with recurrent nocturnal or early morning chest pain following coronary stent implantation, and the electrocardiogram findings were consistent with Wellens' syndrome. Due to a low baseline heart rate, the patient could not tolerate diltiazem, and the response to standard anti-spastic medical therapy was poor. Intracoronary ergonovine provocation testing induced severe spasms of the left anterior descending and left circumflex arteries and reproduced the patient's chest pain. Given the typical presentation of heightened vagal tone, catheter ablation of cardiac autonomic ganglia was performed under the guidance of a three-dimensional electroanatomical mapping system. Post-ablation, repeat ergonovine testing showed markedly attenuated coronary artery spasm that resolved spontaneously, and the patient experienced no chest pain. At the 2-month follow-up, angina had completely resolved. This case suggests that cardiac autonomic ganglion ablation may be a valuable non-pharmacological treatment option for patients with refractory vasospastic angina after PCI who exhibit heightened vagal tone.

## 1 Introduction

Coronary artery spasm is a transient, reversible subtotal or total occlusion caused by excessive constriction of the coronary arteries, serving as a key etiology for myocardial ischemia with non-obstructive coronary arteries. It is primarily characterized by typical vasospastic symptoms, evidence of myocardial ischemia during spontaneous attacks, and definitive diagnostic evidence of coronary artery spasm. Calcium channel blockers are currently the most effective treatment regimen for variant angina, and nitrates can be considered as an adjunctive therapy. However, approximately 20% to 30% of patients progress to refractory variant angina due to poor response or intolerance to pharmacological treatment (1–3). Recent studies have demonstrated that the imbalance of the cardiac autonomic nervous system plays a crucial role in the disease course of coronary artery spasm. Cardioneuroablation (CNA) is a minimally invasive procedure that uses a catheter to target specific nerve clusters on the epicardial surface, known as ganglionated plexi (GP). These plexuses serve as important centers for autonomic control, consisting primarily of parasympathetic neurons. To date, CNA has demonstrated its neuromodulatory value and a favorable safety profile in vasovagal syncope and functional bradyarrhythmias (4–7). Clinical case reports have indicated that CNA can serve as an effective potential intervention for refractory variant angina (8). However, its therapeutic efficacy has not been demonstrated in patients with refractory variant angina secondary to PCI. This article reports a case of a patient with post-PCI refractory variant angina accompanied by typically elevated vagal tone, who achieved favorable outcomes following CNA treatment, aiming to highlight this potential therapeutic option for similar patients.

## 2 Case report

### 2.1 Clinical data

A 45-year-old male patient was admitted due to recurrent chest tightness and chest pain for over 2 years, which had exacerbated for the past 2 days. More than 2 years ago, the patient developed episodic chest pain without apparent triggers. The pain presented as a dull ache, predominantly occurring at night and in the early morning, lasting for several minutes per episode, and was accompanied by distending back pain. On October 8, 2024, the patient underwent coronary angiography and intravascular ultrasound (IVUS). The results revealed no significant stenosis in the left main (LM) coronary artery, with a TIMI flow grade 3. The left anterior descending (LAD) artery exhibited 70% stenosis in the proximal segment, 40%–50% stenosis in the mid segment, and 50% stenosis in the proximal diagonal branch, maintaining a TIMI flow grade 3 (IVUS demonstrated visible thrombus in the proximal LAD, with a minimum lumen area of 3.12 mm^2^ and a plaque burden of 70%. The proximal diagonal branch showed a minimum lumen area of 3.16 mm^2^ and a plaque burden of 48%). The left circumflex (LCX) artery showed no significant stenosis, with a TIMI flow grade 3. The right coronary artery (RCA) revealed approximately 60%–70% stenosis in the mid segment, with a TIMI flow grade 3 (IVUS indicated a minimum lumen area of 5.87 mm^2^ and a plaque burden of 60% in the mid RCA). Two stents were implanted in the LAD.

Postoperatively, the patient regularly took medications including enteric-coated aspirin, ticagrelor, and rosuvastatin calcium. However, the chest pain persisted, presenting with the same characteristics and location as before, and was accompanied by diaphoresis and back pain during severe episodes. A follow-up coronary angiography on April 29, 2025, revealed no significant stenosis in the LM, with TIMI flow grade 3. Visible stents in the proximal-to-mid LAD were patent with no in-stent restenosis and TIMI flow grade 3. There was no significant stenosis in the LCX, with TIMI flow grade 3. Additionally, approximately 50%–60% stenosis was observed in the proximal-to-mid RCA, with TIMI flow grade 3.

### 2.2 Auxiliary examination

The resting electrocardiogram (ECG) revealed inverted or flattened T waves in leads V4-V6. During the episodes of chest pain, ST-segment elevation was observed in leads V2 and V3, along with a symmetrical, upright pseudonormalization of T waves in leads V4-V6 (Figure 1). The chest pain was considered to be induced by coronary artery spasm.

**Figure 1:**
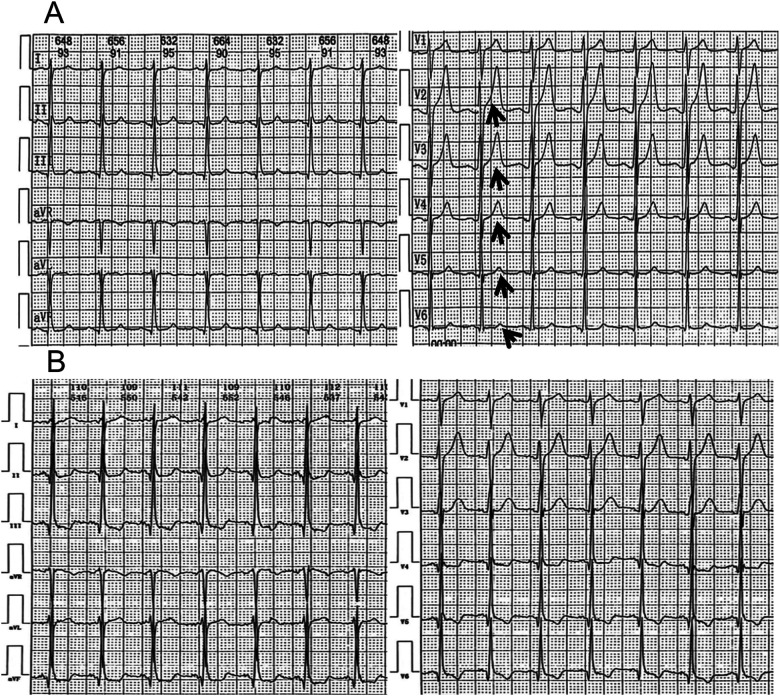
Comparison of 12-lead electrocardiograms during an attack and the resolution of chest pain. **(A)** During the onset of chest pain, T waves in leads V4–V6 exhibited pseudonormalization characterized by symmetrical and upright morphology (indicated by arrows); **(B)** during the asymptomatic period, T waves in leads V4–V6 appeared inverted or flattened.

Due to a slow baseline heart rate and the presence of ventricular escape beats and an escape rhythm on the ambulatory electrocardiogram, the patient was intolerant to diltiazem hydrochloride for anti-vasospastic therapy. Consequently, isosorbide mononitrate was added to the treatment regimen; however, the patient continued to experience episodes of chest pain.

The patient had no prior history of hypertension or diabetes mellitus, but reported a history of smoking and alcohol consumption. Upon admission, physical examination and routine laboratory tests, including complete blood count, liver and renal function, electrolytes, and thyroid function, were all unremarkable except for an elevated troponin T level of 33.92 ng/L (reference range: 0–14 ng/L). A 24-hour ambulatory electrocardiogram revealed an average heart rate of 60 beats per minute (bpm), a maximum heart rate of 111 bpm, and a minimum heart rate of 43 bpm.

### 2.3 Ergonovine provocation test

After a 48-hour discontinuation of vasodilators following admission, the patient underwent selective coronary angiography and an ergonovine provocation test. Initial angiography demonstrated no progression of stenosis in the RCA compared to previous findings, no significant stenosis in the LCX, and patency of the previously implanted stent in the LAD. Given that prior ECG and angiographic results suggested left coronary artery (LCA) involvement, 50 µg of ergonovine was selectively injected into the LM. One minute later, the LAD and LCX began to exhibit spastic responses. Approximately 5 min later, the patient developed typical chest pain accompanied by diaphoresis. Left coronary angiography revealed marked spasm in the LCX and the LAD distal to the stent (TIMI flow grade 3). Following an intracoronary injection of 200 µg of nitroglycerin, both the coronary artery spasm and clinical symptoms completely resolved. 50 µg of ergonovine was specifically injected into the RCA, which showed no significant spasm at 1 and 5 min post-injection (Figures 2A–D).

**Figure 2:**
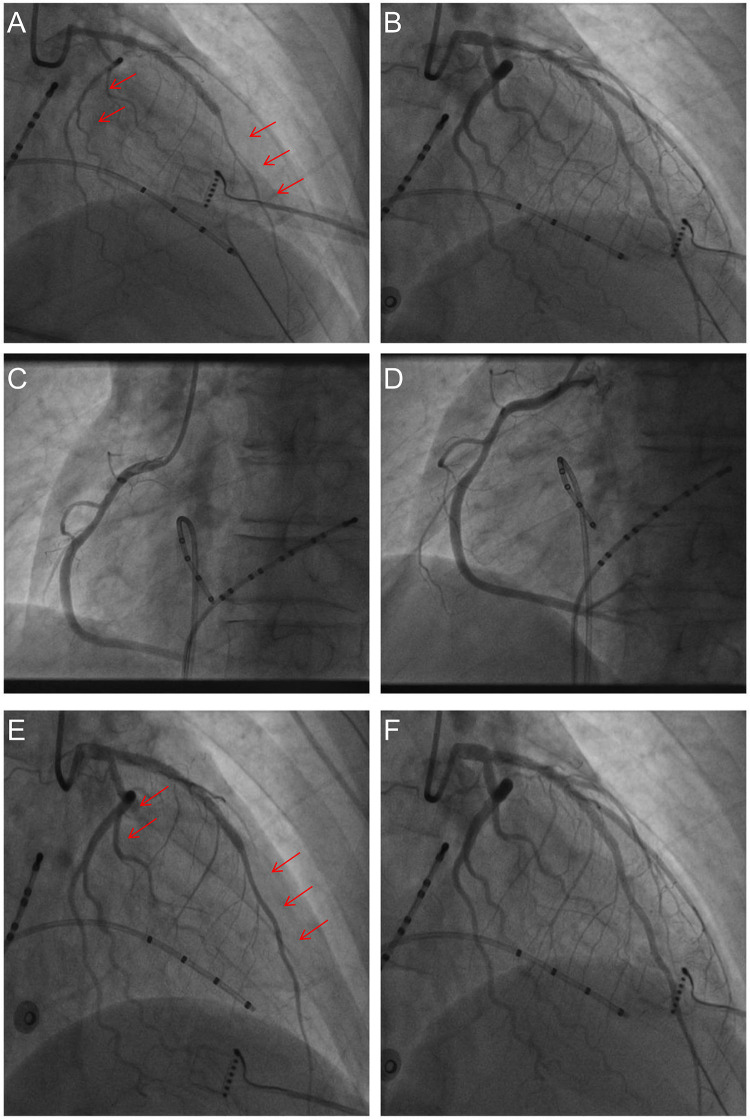
Coronary angiographic images from two ergonovine provocation tests. **(A)** Following intracoronary injection of 50 µg ergonovine into the LCA, severe stenosis was observed in the LAD and LCX (indicated by arrows); **(B)** The vasospasm was completely relieved after intracoronary administration of 200 µg nitroglycerin; **(C)** Intracoronary injection of 50 µg ergonovine into the RCA; **(D)** No significant vasospasm was observed in the RCA. **(E)** Following catheter ablation of the GP, repeated intracoronary injection of 50 µg ergonovine into the LCA induced mild stenosis in the LAD and LCX; **(F)** The mild stenosis in the LAD and LCX resolved spontaneously. LCA, left coronary artery; LAD, left anterior descending artery; LCX, left circumflex artery; RCA, right coronary artery; GP, ganglionated plexi.

### 2.4 Cardiac autonomic ganglionated plexi ablation

Given that the patient's chest pain predominantly occurred at night or in the early morning, accompanied by a slow baseline heart rate, the presence of elevated vagal tone was considered. After obtaining written informed consent from the patient, CNA was performed. A three-dimensional electroanatomical model of the left atrium was constructed using the CARTO 3 system, targeting four major GP: the left superior GP, left inferior GP, right anterior GP, and right inferior GP (Figure 3). The target sites were verified using high-frequency stimulation (20 Hz, 30 mA, pulse width 5 ms, duration 5 s). A vagal response was defined as transient asystole, atrioventricular block, or an increase in the R-R interval of >50%. During the ablation of the left superior GP, a positive vagal response was observed, with the heart rate dropping to a minimum of 40 bpm and the blood pressure decreasing to 90/52 mmHg (Figure 4A). Ablation was performed using an irrigated radiofrequency catheter until the vagal responses were abolished (left superior GP and right anterior GP: power 35 W, ablation index 450; left inferior GP and right inferior GP: power 40 W, ablation index 380). Postoperatively, the sinus heart rate increased to 90 bpm (Figure 4B).

**Figure 3:**
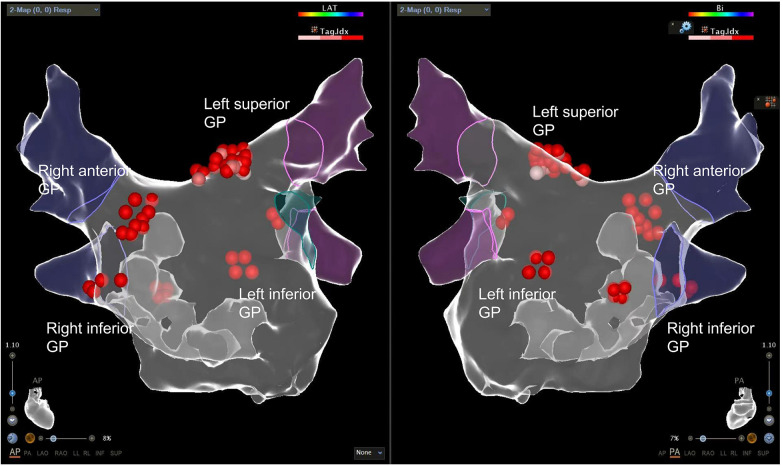
Schematic of catheter ablation targets for cardiac autonomic GP. Three-dimensional electroanatomical maps of the left atrium were reconstructed using the CARTO 3 mapping system. The left panel displays the anteroposterior view, and the right panel displays the posteroanterior view. Red dots represent the ablation sites. GP, ganglionated plexi.

**Figure 4:**
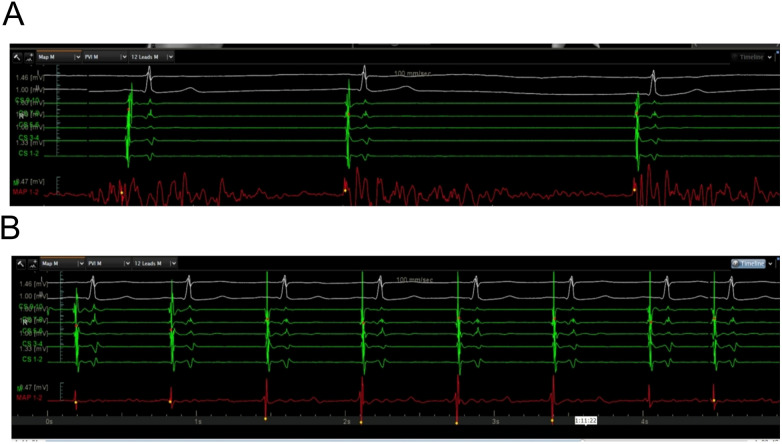
Vagal response and heart rate recovery following left superior GP ablation. **(A)** A positive vagal response was elicited during the ablation of the left superior GP, with the heart rate decreasing to a minimum of approximately 40 bpm; **(B)** post-ablation, the sinus rate increased to 90 bpm. GP, ganglionated plexi.

### 2.5 Postoperative evaluation and follow-up

Following the ablation procedure, a repeat ergonovine provocation test revealed significant changes in the patient's vascular response: the severity of the spasm was attenuated from severe to mild stenosis, and the patient experienced neither chest pain nor diaphoresis during the coronary spasm. Furthermore, the postoperative ergonovine-induced coronary artery spasm resolved spontaneously (Figures 2E,F). Postoperatively, all antispasmodic and vasodilator medications were discontinued, and the patient experienced no further episodes of nocturnal chest pain. Following discharge, the patient was instructed to maintain a symptom diary and immediately record a 12-lead ECG should any chest pain recur. At the 1-month follow-up, the patient reported no angina attacks. Outpatient ambulatory electrocardiogram revealed an average heart rate of 81 bpm, a maximum heart rate of 120 bpm, and a minimum heart rate of 59 bpm, with no arrhythmias, ischemic ST-segment changes, or T-wave changes detected. At the 2-month follow-up, the patient remained completely free of angina episodes.

## 3 Discussion

Wellens syndrome was first described by the Dutch physician Wellens and his colleagues in 1982 (9). It is primarily characterized by T-wave alterations in ECG leads V2 and V3 (occasionally extending to leads V1 and V4–V6) that present during the pain-free interval in patients with a recent history of angina, rather than during the acute angina attack itself. Because this syndrome frequently indicates severe stenosis in the proximal LAD artery and carries a high risk of progressing to an extensive anterior wall myocardial infarction, it is also referred to as LAD coronary T-wave syndrome. Notably, further research has revealed that in addition to severe proximal LAD stenosis, coronary artery spasm can also induce Wellens syndrome (10, 11). The primary diagnostic criteria for Wellens syndrome include a history of angina attacks, normal or minimally elevated cardiac biomarkers, absence of pathologic Q waves in the precordial leads, no ST-segment elevation or ST elevation of less than 1 mm, no loss of R waves in the precordial leads, and biphasic or symmetric deeply inverted T waves in leads V2 and V3 (12).

Subsequently, other investigators have found that Wellens syndrome is not exclusively characterized by biphasic or symmetrically and deeply inverted T waves in leads V2 and V3. In the present case, the patient's episodes predominantly occurred at night or in the early morning, accompanied by typical anginal symptoms (chest pain with diaphoresis). Dynamic electrocardiographic changes were observed between the resting state and the symptomatic episodes. These features are highly consistent with the characteristics of variant angina induced by CAS. Concurrently, the patient's clinical presentation also fulfilled criteria of the aforementioned diagnostic criteria for Wellens syndrome.

Post-PCI coronary artery spasm (CAS) is a significant cause of acute coronary occlusion and in-stent thrombosis. Severe and prolonged CAS can precipitate acute myocardial infarction, arrhythmias, heart failure, and even death. The PCI procedure itself can act as a trigger for CAS (13, 14). A study involving 57 patients with ST-segment elevation myocardial infarction (STEMI) who underwent stent implantation revealed that at 2–5 weeks post-procedure, the incidence of acetylcholine-induced coronary artery spasm was as high as 70.2%, with multivessel spasm accounting for 29.8% of these cases (15). Yoshikawa et al. reported a case in which coronary spasm occurring in a side branch “jailed” after stent implantation resulted in a myocardial infarction. They postulated that stent implantation could induce vascular endothelial dysfunction and local inflammation, an effect that occurs not only at the stent edges but may also extend to the ostium of the side branch covered by the stent (16).

Yamamoto et al. analyzed 1,039 patients and found a significantly higher positive rate of acetylcholine provocation testing in those with a stenting history (60.6%) than in those without (52.0%); coronary spasm incidence was also higher in stented (50.0%) than non-stented vessels (33.3%), and was particularly common at the stent's distal edge, possibly due to stent-induced endothelial stimulation and local vasomotor imbalance (17). In response, Marialucia Milite proposed the “vascular wall injury hypothesis,” suggesting stenting-related mechanical injury (e.g., balloon dilatation) contributes to spasm (18).

Previous studies investigating the mechanisms underlying post-PCI coronary artery spasm have primarily focused on vascular endothelial dysfunction, inflammatory and hypersensitivity reactions, and vascular wall injury. In the present case, the patient's post-PCI chest pain predominantly occurred at rest during the night or early morning. The electrocardiograms during these episodes were consistent with Wellens syndrome, suggesting symptoms of vagal hyperactivity. Based on this, it is considered that the patient's post-PCI coronary spasm is associated with the dual factors of vascular mechanisms and increased vagal tone. Specifically, the prior PCI may have established a vascular substrate of endothelial vulnerability, while vagal hyperactivity provided the trigger for the spasm. Compared with the case report by Lu et al., the present case features refractory coronary spasm secondary to PCI, with the spasm predominantly involving the LAD and LCX rather than the RCA, and accompanied by a Wellens-like ECG pattern, which can provide new incremental clinical value to this field.

The cardiac autonomic nervous system modulates coronary artery tone through a highly complex network (19, 20). Under physiological conditions, acetylcholine released from postganglionic parasympathetic nerve fibers acts on vascular endothelial cells to mediate vasodilation via the nitric oxide pathway. However, in the setting of vascular endothelial dysfunction, acetylcholine can directly access and stimulate M3 muscarinic receptors expressed on vascular smooth muscle. By triggering intracellular calcium release via the Gq protein-coupled signaling pathway, this leads to excessive vascular smooth muscle contraction, thereby precipitating coronary artery spasm (21–23). In this patient, chronic smoking and PCI-induced endothelial dysfunction formed the pathophysiological basis for coronary artery spasm secondary to vagal hyperactivity. Given the poor response to conventional antispasmodic agents, we adopted a therapeutic strategy targeting the upstream regulatory centers of the cardiac autonomic nervous system. GP act as key integration centers for autonomic nerves in epicardial fat pads. GP ablation guided by high-frequency stimulation selectively interrupts vagal innervation, reduces vagal tone, restores autonomic balance, and abolishes vasospasm caused by abnormal vagal activation. Post-procedure, the patient's resting heart rate increased from 40 to 90 bpm, confirming effective vagal blockade. A repeat ergonovine provocation test showed significantly attenuated coronary spasm, which resolved spontaneously. At the 2-month follow-up, the patient's angina symptoms completely resolved. These results suggest that cardiac autonomic GP catheter ablation may serve as a non-pharmacological option for patients with post-PCI refractory variant angina, especially those with increased vagal tone.

However, the present study has certain limitations. It is only a single case report, and large-scale clinical studies to support these findings are currently lacking worldwide. Furthermore, the increased vagal tone in this patient was suggested solely by indirect evidence, such as the timing of symptom onset, baseline bradycardia, and post-ablation heart rate elevation. Objective quantitative parameters and tests, including heart rate variability, heart rate deceleration capacity, time and frequency domain heart rate variability values, the low frequency to high frequency ratio, the atropine test, and sinoatrial and atrioventricular node function tests, were not assessed, making it impossible to quantitatively confirm the modulatory effect of ablation on autonomic balance. In addition, the short follow-up period precluded the evaluation of long-term post-ablation efficacy and the potential risk of recurrence. Consequently, the efficacy of this therapeutic approach warrants further validation through long-term follow-up and larger-scale clinical studies.

## 4 Conclusion

This case report suggests that catheter ablation of CNA may represent a potential non-pharmacological treatment option for selected patients with refractory vasospastic angina following percutaneous coronary intervention, particularly when the clinical presentation is characterized by heightened vagal tone. The patient achieved complete resolution of angina symptoms, exhibited objective improvement in ergonovine-provoked coronary artery spasm, and showed sustained clinical benefit at 2-month follow-up. These findings suggest that modulating the cardiac autonomic nervous system by targeting ganglionated plexi may help restore autonomic balance and reduce vagally triggered coronary spasms, particularly in cases where conventional anti-spastic therapy is poorly tolerated or ineffective. However, given that this evidence is derived from a single case, large-scale clinical trials with extended long-term follow-up are necessary to validate the efficacy, safety, and durability of this interventional approach.

## Data Availability

The raw data supporting the conclusions of this article will be made available by the authors, without undue reservation.

## References

[1] Khan A Lahmar A Riasat M Ehtesham M Asif H Khan W. Myocardial infarction with non-obstructive coronary arteries: an updated overview of pathophysiology, diagnosis, and management. Cureus. (2022) 14:e23602. 10.7759/cureus.23602 35505697 PMC9053360

[2] Ralota KK Layland J. Vasospastic angina: pathophysiology, diagnosis, and emerging therapeutic approaches. World J Cardiol. (2025) 17:108594. 10.4330/wjc.v17.i10.108594 41181655 PMC12576579

[3] Jenkins K Pompei G Ganzorig N Brown S Beltrame J Kunadian V. Vasospastic angina: a review on diagnostic approach and management. Ther Adv Cardiovasc Dis. (2024) 18:17539447241230400. 10.1177/17539447241230400 38343041 PMC10860484

[4] Li C Hu Y Li Y Zeng Z Yu W Zhou Z. Effects of cardioneuroablation for vasovagal syncope: ganglionated plexus localization by tentative anatomical ablation and high-frequency electrical stimulation. BMC Cardiovasc Disord. (2025) 25:505. 10.1186/s12872-025-04933-z 40660118 PMC12257755

[5] Zuhair M Keene D Panagopoulos D Malcolme-Lawes L Porter B Kanagaratnam P. Catheter ablation for vasovagal syncope: the therapeutic potential of gateway plexi. Arrhythm Electrophysiol Rev. (2025) 14:e01. 10.15420/aer.2024.36 39981423 PMC11836605

[6] Armani Prata A Katsuyama E Scardini P Antunes V Granja J Coan AC. Cardioneuroablation in patients with vasovagal syncope: an updated systematic review and meta-analysis. Heart Rhythm. (2025) 22:526–35. 10.1016/j.hrthm.2024.07.103 39067734

[7] Aksu T Brignole M Calo L Debruyne P Di Biase L Deharo JC. Cardioneuroablation for the treatment of reflex syncope and functional bradyarrhythmias: a scientific statement of the European Heart Rhythm Association (EHRA) of the ESC, the Heart Rhythm Society (HRS), the Asia Pacific Heart Rhythm Society (APHRS) and the Latin American Heart Rhythm Society (LAHRS). Europace. (2024) 26:euae206. 10.1093/europace/euae206 39082698 PMC11350289

[8] Fan D Guo Z He B Hu Y Lu Z. Cardioneuroablation for coronary artery vasospasm: a case report. Eur Heart J Case Rep. (2025) 9:ytaf456. 10.1093/ehjcr/ytaf456 41050530 PMC12492480

[9] Wellens HJJ Bär FWHM Lie KI. The value of the electrocardiogram in the differential diagnosis of a tachycardia with a widened QRS complex. Am J Med. (1978) 64:27–33. 10.1016/0002-9343(78)90176-6 623134

[10] Abulaiti A Aini R Xu H Song Z. A special case of Wellens’ syndrome. J Cardiovasc Dis Res. (2013) 4:51–4. 10.1016/j.jcdr.2013.02.016 24023474 PMC3758090

[11] Diallo TH Djafarou Boubacar R Azday IS Fellat R Fellat N. Urgent percutaneous coronary intervention in type 2 Wellens’ syndrome: a case report of an atypical presentation in an elderly patient. SAGE Open Med Case Rep. (2024) 12:2050313X241271771. 10.1177/2050313X241271771 39650170 PMC11622294

[12] Alexander J Rizzolo D. Wellens syndrome: an important consideration in patients with chest pain. JAAPA. (2023) 36:25–9. 10.1097/01.JAA.0000911188.18646.31 36701576

[13] Ichimoto E Kobayashi T Kato M Kawana H Hasegawa A Miyahara H. Vasospastic angina diagnosed after percutaneous coronary intervention. J Hosp Gen Med. (2024) 6(5):120–5. 10.60227/jhgmeibun.6.5_120

[14] Crea F. Angina due to coronary artery spasm after a percutaneous coronary intervention. Eur Heart J Suppl. (2019) 21:G30–1. 10.1093/eurheartj/suz200 31736671 PMC6849460

[15] Katoh D Mizuno Y Harada E Ito T Morikawa Y Nakagawa H. High incidence of provoked coronary spasm in the presence of a stent after myocardial infarction: therapeutic and prognostic implications. Coron Artery Dis. (2012) 23:141–5. 10.1097/MCA.0b013e32835115ee 22327063

[16] Yoshikawa H Sugiyama T Araki M Yonetsu T Sasano T. Acute myocardial infarction caused by vasospasm of a jailed diagonal branch subsequent to stent implantation in the left anterior descending artery: a case report. Eur Heart J Case Rep. (2024) 8:ytae421. 10.1093/ehjcr/ytae421 39176023 PMC11339709

[17] Yamamoto K Saito Y Tateishi K Kato K Kitahara H Kobayashi Y. Relation between previously implanted coronary stents and vasospasm: patient- and vessel-level analysis. Int J Cardiol. (2025) 431:133248. 10.1016/j.ijcard.2025.133248 40209940

[18] Kubik S. Relation between previously implanted coronary stents and vasospasm: do we modulate the risk of post-procedural vasospasm by exacerbating vessel wall injury? Int J Cardiol. (2025) 437:133461. 10.1016/j.ijcard.2025.133461 40482825

[19] Ziegler KA Engelhardt S Carnevale D McAlpine CS Guzik TJ Dimmeler S. Neural mechanisms in cardiovascular health and disease. Circ Res. (2025) 136:1233–61. 10.1161/CIRCRESAHA.125.325580 40403111

[20] Sato T Hanna P Mori S. Innervation of the coronary arteries and its role in controlling microvascular resistance. J Cardiol. (2024) 84:1–13. 10.1016/j.jjcc.2024.01.005 38346669 PMC12533524

[21] Benabou L Vaseghi M. Cardiac sympathetic innervation and ventricular arrhythmias in structural heart disease: current peripheral neuromodulation therapies and emerging therapeutic targets. Arrhythm Electrophysiol Rev. (2025) 14:e34. 10.15420/aer.2025.24 41524080 PMC12784272

[22] SenthilKumar G Limpert RH Lindemer BJ Freed JK. Acetylcholine-Induced dilation in human arterioles requires ceramide formation. ATVB. (2024) 44:1895–7. 10.1161/ATVBAHA.124.320916 PMC11268997 38934116

[23] Varzideh F Jankauskas SS Mone P Kansakar U Santulli G. Autonomic neurotransmission in cardiovascular regulation and pathophysiology. Front Neurosci. (2026) 19:1739330. 10.3389/fnins.2025.1739330 41668724 PMC12883788

